# Impact of Lhcx2 on Acclimation to Low Iron Conditions in the Diatom *Phaeodactylum tricornutum*

**DOI:** 10.3389/fpls.2022.841058

**Published:** 2022-03-16

**Authors:** Jochen M. Buck, Marie Wünsch, Alexander F. Schober, Peter G. Kroth, Bernard Lepetit

**Affiliations:** Plant Ecophysiology, Department of Biology, University of Konstanz, Konstanz, Germany

**Keywords:** diatoms, *Phaeodactylum tricornutum*, iron, NPQ, photoprotection, Lhcx, reactive oxygen species, photosystem

## Abstract

Iron is a cofactor of photosystems and electron carriers in the photosynthetic electron transport chain. Low concentrations of dissolved iron are, therefore, the predominant factor that limits the growth of phototrophs in large parts of the open sea like the Southern Ocean and the North Pacific, resulting in “high nutrient–low chlorophyll” (HNLC) areas. Diatoms are among the most abundant microalgae in HNLC zones. Besides efficient iron uptake mechanisms, efficient photoprotection might be one of the key traits enabling them to outcompete other algae in HNLC regions. In diatoms, Lhcx proteins play a crucial role in one of the main photoprotective mechanisms, the energy-dependent fluorescence quenching (qE). The expression of Lhcx proteins is strongly influenced by various environmental triggers. We show that Lhcx2 responds specifically and in a very sensitive manner to iron limitation in the diatom *Phaeodactylum tricornutum* on the same timescale as the known iron-regulated genes *ISIP1* and *CCHH11.* By comparing Lhcx2 knockout lines with wild type cells, we reveal that a strongly increased qE under iron limitation is based on the upregulation of Lhcx2. Other observed iron acclimation phenotypes in *P. tricornutum* include a massively reduced chlorophyll *a* content/cell, a changed ratio of light harvesting and photoprotective pigments per chlorophyll *a*, a decreased amount of photosystem II and photosystem I cores, an increased functional photosystem II absorption cross section, and decoupled antenna complexes. H_2_O_2_ formation at photosystem I induced by high light is lowered in iron-limited cells, while the amount of total reactive oxygen species is rather increased. Our data indicate a possible reduction in singlet oxygen by Lhcx2-based qE, while the other iron acclimation phenotype parameters monitored are not affected by the amount of Lhcx2 and qE.

## Introduction

Photosynthetic organisms have a high demand for iron due to the utilization of iron as a cofactor of redox complexes and electron carriers in the mitochondrial respiratory and plastid photosynthetic electron transport chain. In the oceans, iron is distributed unequally (Behrenfeld et al., 2009). Coastal regions are saturated with iron due to various influxes, e.g., upwelling from the soil, introduction by rivers, and atmospheric input (Duce and Tindale, 1991; Jickells et al., 2005). In major parts of the open ocean and, especially, in the southern hemisphere, the situation is completely different: Upwelling of iron from the deep sea as well as introduction by rivers is lacking. Furthermore, the atmospheric iron input rate is rather low and is reduced gradually, depending on the distance from the coasts and, especially, from dust-generating deserts (Duce and Tindale, 1991; Jickells et al., 2005; Behrenfeld et al., 2009; Smith et al., 2016). These oceanic areas are termed “high nutrient–low chlorophyll” (HNLC) zones, because they are characterized by a low phytoplankton density despite high nitrate and phosphate availability (Martin et al., 1994). Diatoms are relatively abundant in these regions compared to other photosynthetic organisms and reach very high densities once iron is re-introduced (Boyd et al., 2000; Gómez et al., 2007). This has been impressively shown by iron fertilization experiments to HNLC regions, which resulted in blooming of mainly diatom species and proved that iron is the limiting growth factor (Martin et al., 1994; Boyd et al., 2000; Gervais et al., 2002; Boyd et al., 2007). Therefore, revealing the special adaptations and survival strategies of diatoms to iron limitation is of particular interest. Diatoms possess multiple strategies to cope with low iron availability, which include: (i) replacing iron-containing proteins by alternative proteins, e.g., cytochrome c_6_ by plastocyanin and ferredoxin by flavodoxin (Behrenfeld and Milligan, 2012); (ii) intracellular recycling of iron and efficient iron uptake from the environment (Maldonado et al., 2001; Kustka et al., 2007; Smith et al., 2016; McQuaid et al., 2018); (iii) downregulation of total photosynthetic capacity (Allen et al., 2008; Behrenfeld and Milligan, 2012; Smith et al., 2016); (iv) reduction of iron demanding mitochondrial respiratory complexes (Bozzato et al., 2021). The genes encoding for some proteins like iron starvation responsive proteins (ISIPs) or the transcription factor CCHH11 are highly upregulated under iron limitation (Allen et al., 2008; Smith et al., 2016), with ISIP1 being involved in a high-affinity uptake mechanism for iron complexes (Kazamia et al., 2018).

Characteristic for HNLC regions is a reduced photosynthetic yield of photosystem II [Y(PSII)] (Gervais et al., 2002; Behrenfeld et al., 2006; Behrenfeld et al., 2009). This reduction in Y(PSII) is also observed in the diatom *Phaeodactylum tricornutum* under iron starvation (Greene et al., 1991; Allen et al., 2008), but not during additional parallel macronutrient starvation (Behrenfeld et al., 2006). The reduced Y(PSII) is supposed to be the result of: (i) a pool of chlorophyll-binding proteins, which are not coupled to (functional) reaction centers and which, therefore, increase the background fluorescence (F_o_), and (ii) a massively reduced capacity of the electron transport chain due to reduction of the cytb_6_f complex, cytochrome c_6_, and PSI. This leads to a more reduced PQ pool even under dark conditions (Greene et al., 1991; Morales et al., 2001; Behrenfeld et al., 2006; Roncel et al., 2016; Smith et al., 2016). Interestingly, the remaining photosystem II (PSII) complexes in *P. tricornutum* enhance their functional absorption cross section in response to iron limitation (Greene et al., 1991; Alderkamp et al., 2012; Trimborn et al., 2019), While the total antenna protein expression and the amount of photosynthetic pigments per cell are only moderately affected (Greene et al., 1991; Greene et al., 1992; Allen et al., 2008; Smith et al., 2016).

In nature, supersaturating light can result in the formation of potentially toxic quantities of reactive oxygen species (ROS) (Strzepek and Harrison, 2004; Behrenfeld and Milligan, 2012). This effect may be especially prominent in iron-limited cells due to their reduced electron turnover capacity. At Photosystem I (PSI), superoxide, hydrogen peroxide (H_2_O_2_), and the hydroxyl radical are ROS species that are formed as intermediates during the reduction of oxygen to water in the Mehler reaction (Asada, 2000; Nishiyama et al., 2006). At PSII, triplet chlorophyll *a* (^3^Chl *a*) is formed *via* charge recombination or direct intersystem crossing of excited singlet Chl *a*, which–*via* reaction with triplet oxygen–causes formation of singlet oxygen (^1^O_2_) (Nishiyama et al., 2006; Krieger-Liszkay et al., 2008; Triantaphylidès and Havaux, 2009). Therefore, light protection mechanisms might be essential to reduce light-induced cellular damages under iron limitation.

Energy-dependent fluorescence quenching (qE), a subtype of non-photochemical fluorescence quenching (NPQ), is one of the main photoprotective mechanisms that allow the harmless dissipation of absorbed energy as heat (Lavaud and Goss, 2014; Goss and Lepetit, 2015). qE is regulated by the pH gradient generated across the thylakoid membrane (ΔpH), and, in diatoms, involves the xanthophyll cycle pigment diatoxanthin (Dt) produced from diadinoxanthin (Dd) as well as Lhcx proteins (Lavaud et al., 2002a,b; Goss et al., 2006; Bailleul et al., 2010; Taddei et al., 2016; Buck et al., 2019, 2021). In *P. tricornutum*, three Lhcx isoforms are involved in qE (Buck et al., 2019). While Lhcx1 mediates a basal qE capacity (Bailleul et al., 2010; Buck et al., 2019), *Lhcx2* and *Lhcx3* are upregulated under high light, which goes along with a rise in qE capacity (Lepetit et al., 2017). Besides high light, iron limitation also induces the expression of Lhcx2 and, therefore, was assumed to regulate qE in this condition (Taddei et al., 2016).

In this work, we used Lhcx2 knockout strains of *P. tricornutum* to investigate the impact of Lhcx2 on various physiological parameters under iron limitation. Without Lhcx2, *P. tricornutum* can upregulate qE capacity under iron starvation only to a limited extent. The knockout of Lhcx2 does not affect several other observed physiological parameters besides qE capacity and neither seems to be involved in the reduced photosynthetic yield and uncoupling of antennae nor in the pigmentation changes that occur under iron limitation. The total amount of ROS induced by high light is enhanced in iron-limited cells, albeit with reduced H_2_O_2_ levels, thus indicating an enhanced formation of ^1^O_2_ instead of H_2_O_2_. Here, our data indicate a putative role of qE in reducing total ROS by reducing excitonic pressure in PSII.

## Materials and Methods

### Culture Conditions

*Phaeodactylum tricornutum* (UTEX646; Pt4) was grown in white low light (∼40-μmol photons m^–2^ s^–1^, L58W/30 Osram, Germany) with 16 h of light and 8-h-darkness cycles at 20°C. Cultures were kept in a Provasoli’s-enriched F/2 medium (Guillard, 1975) using 16.6-g L^–1^ artificial sea salt (Tropic Marin CLASSIC; Dr. Biener GmbH, Wartenberg, Germany).

### CRISPR/Cas9-Based Gene Knockout of Lhcx2 (x2KO_b)

About 20-bp single guide RNAs (sgRNA) featuring a + NGG Protospacer Adjacent Motif (PAM) for *Lhcx2* [Phatr2_54065;chr_1:2471232-2472285(+)] were predicted using the CRISPOR web server^1^ (Concordet and Haeussler, 2018). Off-target analysis was performed with the tool’s built-in off-target prediction by choosing “*Phaeodactylum tricornutum* EnsemblProtists 79 (ASM15095v2_bd)” as a reference genome. We selected the sgRNA “CTGGTTGAGGTGAGTAATGG” as it had no predicted genomic off targets and binds within the first exon of *Lhcx2* and featured high “Doench 16” and “Moreno-Mateos” scores of both 60, while “Out-of-Frame” and “Lindel” scores were 68 and 77, respectively. The construction of the episomal vector for the CRISPR/Cas9-mediated knockout of Lhcx2 precisely followed the protocol by Nymark et al. (2017). In short, 24 bp complementary oligonucleotides with 5′ TCGA and ACCC overhangs were hybridized. The resulting sgRNA-encoding dsDNA was ligated to a *Bsa*I-linearized and gel-purified bacterial conjugation vector pPtpuc3_diaCas9_sgRNA (Sharma et al., 2018) before it was transformed to Escherichia *coli* XL1blue. The readily cloned pPtpuc3_diaCas9_sgRNA (Lhcx2_386rev) cargo vector was transformed in *E. coli* DH10β cells, carrying the mobilization helper plasmid pTA-Mob (Strand et al., 2014). Cells carrying this helper plasmid are competent to mediate delivery of oriT-containing plasmids toward *P. tricornutum*. Resulting DH10β cells were used to transform *P. tricornutum* by following the procedure of Karas et al. (2015), considering all modifications suggested by Diner et al. (2016).

We isolated the genomic DNA of potential mutants with the Nexttec 1-step DNA isolation kit (Biozym, Hessisch Oldendorf, Germany). The gene region surrounding the binding site for the CRISPR-Cas construct was amplified *via* allele-specific PCR using HiDi polymerase (MyPols, Konstanz, Germany) and primers with allele-specific binding of the 3′ end, designed in Buck et al. (2019). PCR products were sequenced (Microsynth, Balgach, Switzerland) and showed deletions of 22 bp in one allele and of 1 bp in the second allele in case of x2KO_b. Sequences of both alleles of x2KO_b can be found in Supplementary Figure 1.

### Iron Limitation Experiments

A custom salt mixture was prepared, which contained 12.25-g L^–1^ NaCl, 5.55-g L^–1^ MgCl_2_. 6 H_2_O, 2.05-g L^–1^ Na_2_SO_4_, 0.77-g L^–1^ CaCl_2_. 2 H_2_O, 0.35-g L^–1^ KCl, 0.1-g L^–1^ KBr, 0.015-g L^–1^ H_3_BO_4_, and 0.0015-g L^–1^ NaF. Vitamin and nutrient solutions analogous to the F/2 medium were added, while due to its coverage of additional metals, L1 trace metals were prepared according to Guillard and Hargraves (1993) without addition of iron. All other cultivation conditions (temperature, light intensity, day/night rhythm) were as described in Section “Culture Conditions.” For inoculation of the iron-starvation experiments, nutrient replete cells in the late exponential phase were used. The cells were washed three times, counted using a Coulter Counter (Beckman Coulter, Brea, CA, United States) and inoculated in a density of 10^5^ cells ml^–1^. The cultures were regularly diluted to about 10^6^ cells ml^–1^, keeping them all the time saturated with all nutrients except iron. Controls were proceeded the same way, but iron solution was added in the same concentration like the F/2 medium.

### Western Blots

Western blots were performed according to Buck et al. (2019). In short, cells were homogenized in a lysis buffer [50-mm Tris–HCl pH 6.8, 2% SDS, and 1× protease inhibitor (cOmplete; Roche)] with a cell homogenizer (Savant Fastprep FP120; Thermo Fisher) and glass beads. Supernatants according to 1 μg of Chl *a* were separated on 14% LDS–polyacrylamide gels, blotted on nitrocellulose membranes, blocked with 1× Rotiblock (Carl Roth, Karlsruhe, Germany) and incubated with either an anti-Rubisco antibody (AS03037; Agrisera, Vännäs, Sweden) or an anti-Lhcx antibody, each in 1:10,000 dilution [raised by Buck et al. (2019); AS194367; Agrisera] in 1× Rotiblock. The secondary HRP-labeled goat anti-rabbit antibody (AS09602; Agrisera) was either applied in 1:10,000 (Lhcx) or 1:20,000 (Rubisco) dilution in 1× Rotiblock. Detection was performed in an Odyssey FC (LI-COR) with Roti-Lumin Plus (Carl Roth) as substrate.

### Fluorescence Measurements

Fluorescence measurements were performed using a DUAL-PAM (Walz, Effeltrich, Germany) in case of 10-min high-light experiments or an Imaging-PAM (Walz, Effeltrich, Germany) in case of iron depletion experiments. Kinetic measurements with the DUAL-PAM were performed with low light-acclimated cells in density of 10-μg Chl *a* ml^–1^ to which 4-mM NaHCO_3_ was added. The DUAL-PAM actinic light was set to similar proportions of red and blue lights. The cells were exposed to high light (1,700-μmol photons m^–2^ s^–1^) for 10 min, followed by a low light recovery phase (40-μmol photons m^–2^ s^–1^) for 18 min. The fluorescence value obtained with the first saturating light flash (8,000-μmol photons m^–2^ s^–1^) after the onset of the actinic light was used as reference (F_m_) for the calculation of NPQ, using the Stern–Volmer equation NPQ = F_m_/F_m_′ − 1. Kinetic and F_v_/F_m_ measurements in the Imaging-PAM were performed with cell concentrations of similar Fo intensities (1–2-μg Chl *a* ml^–1^). The lowest possible measuring light was used for determination of F_o_ and a light flash of 2,400-μmol photons m^–2^ s^–1^ for determination of F_m_. The NPQ capacity was determined by taking F_m_’ obtained by the last flash after 10-min exposure to ∼500-μmol photons m^–2^ s^–1^ of blue light.

### Functional Absorption Cross Section Measurements

Functional absorption cross sections were measured as described in detail in Buck et al. (2019) using a DUAL-PAM 100 (Walz, Effeltrich, Germany). In short, low-light acclimated cells were adjusted to 10-μg Chl *a* ml^–1^, and initial F_o_ was determined. The cells were treated with 10-μM DCMU to block electron flow between PSII and PSI and exposed to a short red-light flash of low intensity, inducing a rise in fluorescence, reaching F_m_. To compensate the DCMU-induced rise in F_o_, the initial linear slope in fluorescence within the first 5 ms was prolonged to the initial F_o_ value of the untreated cells. Cross sections were calculated according to the procedure proposed by Tian et al. (2019) by extrapolation of the recorded fluorescence increase in DCMU-treated cells to the initial F_o_ value of untreated cells. The extrapolated fluorescence curves were then normalized to values between 0 and 1 to calculate the reciprocal area above the curves, representing the functional absorption cross section of PSII.

### 77 K Fluorescence Emission

For low-temperature fluorescence emission spectra, cells were concentrated to 2.5-μg Chl *a* ml^–1^ in a standard F/2 medium. As a fluorescence standard, His-tagged Superfolding GFP-His6 (sfGFP) was added to the samples. sfGFP was overexpressed in *E. coli* BL21 DE3 pLysS Rosetta cells and purified. The vector pET28a-sfGFP (Peeler and Mehl, 2012) was transformed *via* heat shock into *E. coli* cells. Overexpression was induced with 1-mM IPTG at 23°C overnight. sfGFP was purified from the lysates by Ni-NTA (Thermo Fisher; Waltham, MA, United States) in ECONO gravity columns (BioRad; Hercules, CA, United States). sfGFP was added in the same concentration to each algal sample, which gave fluorescence signals in a similar intensity level to the Chl *a* fluorescence at 686 nm. Samples were frozen in liquid nitrogen in a 1-ml plastic cuvette. Measurements were performed in a Jobin Yvon Fluoromax 4 (Horiba; Kyoto, Japan) using an excitation wavelength of 472 nm with a slit width of 2 nm. Emission was detected between 485 and 750 nm with a slit width of 2 nm, and the maximum of sfGFP emission at 77K was detected at around 493 nm.

### Pigment Analysis by HPLC

Pigments were analyzed by HPLC according to Jakob et al. (1999) using the same procedure described by Buck et al. (2019). Low-light grown cells under iron-replete and iron-depleted conditions were harvested on 1.2-μm polycarbonate filters (Millipore, Burlington, MA, United States), frozen in liquid nitrogen, stored at -80°C, and the pigments were extracted in 81% methanol/9% 0.2-M ammonium acetate/10% ethyl acetate (v/v/v). Additionally, the cells grown in iron-replete conditions were concentrated to 10-μg Chl *a* ml^–1^ and supplemented with 4-mM NaHCO_3_. They were then exposed to 2 h of high light, followed by a 30-min recovery period in the Dual-PAM as specified in section “Fluorescence Measurements,” and pigments before high light, after 2 h of high light exposure, and after 30 min of recovery were harvested and extracted as described above. To monitor the xanthophyll cycle pigment amount during blocking translation of nuclear-encoded genes, pigments were also extracted from cells that were inoculated with 2-μg ml^–1^ cycloheximide prior to high light illumination and then exposed to the same light conditions. The extracted pigments were analyzed in a LaChrom Elite HPLC system (VWR-Hitachi) equipped with a Nucleosil 120-5 C18 column (Macherey-Nagel, Düren, Germany) using a linear gradient of two eluents [90% MeOH/10% 0.5-M NH_4_Ac (v/v) and 90% MeOH/10% ethyl acetate (v/v)]. The pigments were quantified using previously determined calibration factors.

### Determination of Hydrogen Peroxide Formation With Amplex Red

For determination of cellular H_2_O_2_ concentrations, the highly specific Amplex™ Red Hydrogen Peroxide/Peroxidase Assay kit (Thermo Fisher, Waltham, MA, United States) was applied. Either cells in their exponential growth phase in a concentration according to ∼1–3-μg Chl *a* ml^–1^ or cells after the 10-day iron limitation experiments were used. The cells were concentrated to 5-μg Chl *a* ml^–1^ in a 50-mM phosphate buffer pH 7.4 and treated with Amplex Red in a final concentration of 10 μM. NaHCO_3_ was added in a final concentration of 2-μM to avoid CO_2_ limitation during experiments. For DCMU experiments, DCMU was added in a final concentration of 5-μM before the illumination period. The cultures were transferred into black 96-well plates with an optical bottom (Nunc™ MicroWell™; Thermo Fisher, Waltham, MA, United States). Illumination with 500-μmol photons m^–2^ s^–1^ was provided using an LED light panel (ASTIR 5000K; Bioledex, Augsburg, Germany) and a rose-pink filter (002; LeeFilters, Hampshire, United Kingdom) to avoid bleaching of the fluorescent reaction product. The cells of all experimental setups were incubated for the same time with Amplex Red but kept shorter in the light, e.g., 30-min treated cells had been incubated for 30 min in darkness before illumination. Fluorescence signals were measured by an infinite 200Pro (Tecan, Männedorf, Switzerland) or a SpectraMax iD3 (Molecular Devices, San José, CA, United States) plate reader with 530-nm excitation and 585/590-nm emission. Because slight spontaneous oxidation of Amplex Red occurs during the measurements even without light exposure (Zhou et al., 1997), the Amplex Red signals of non-illuminated controls of the same experimental setup were subtracted from the illuminated ones, followed by normalization of each experimental setup to the wild type fluorescence at 60-min illumination.

### Determination of General Oxidative Stress With CellROX Orange

For determination of the total amount of reactive oxygen species, the CellROX Orange kit (Thermo Fisher, Waltham, MA, United States) was used according to the manufacturer’s instructions. The cells were adjusted to 5-μg Chl *a* ml^–1^ in a 50-mM phosphate buffer pH 7.4 and supplemented with NaHCO_3_ (2-μM final concentration). CellROX Orange was added to the cells in a final concentration of 25 μM. For DCMU experiments, 5-μM DCMU had been added before the illumination period. The cells were illuminated in the same way like in the Amplex Red experiments. Fluorescence signals were determined by a SpectraMax iD3 plate reader (Molecular Devices, San José, CA, United States) and 535-nm excitation and 575-nm emission wavelengths. The light-induced oxidative stress was calculated the same way described for Amplex Red. However, as CellROX also developed a significant signal over time during illumination in a medium alone, we refrained from determining CellROX at two different time points but rather compared all strains at time point 60-min high light exposure.

## Results

### Investigation of Non-photochemical Fluorescence Quenching and Xanthophyll Cycle Dynamics Under Iron-Replete Conditions

In our experiments, we used *P. tricornutum* (Pt4) wild type, two Pt4 Lhcx2 knockout strains (x2KO_a and x2KO_b), as well as a complemented x2KO_a strain (x2KO_a + x2). The x2KO_a and x2KO_a + x2 strains have been previously produced *via* TALEN and characterized under light stress conditions by Buck et al. (2019). The x2KO_b was newly produced in this study *via* a CRISPR-Cas-based approach (cf. Supplementary Figure 1 for sequence information).

**FIGURE 1:**
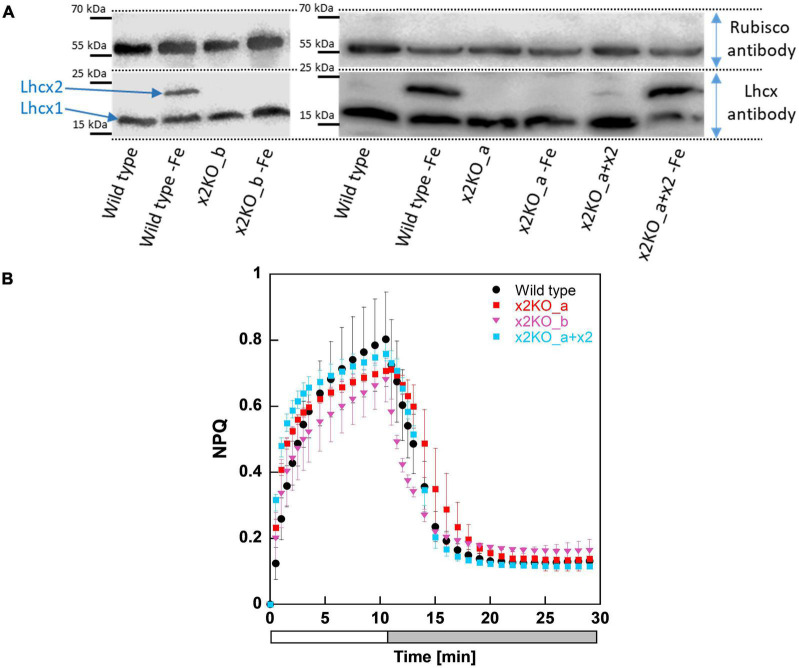
Western blots and non-photochemical fluorescence quenching (NPQ) measurements of *Phaeodactylum tricornutum* wild type and Lhcx2 mutant lines. **(A)** Western blots under iron-limited and iron-replete conditions. Cells were harvested 7 days after inoculation to iron depleted and replete mediums and were kept under low light of 30–40-μmol photons m^–2^ s^–1^. The lower part of the blot was incubated with an Lhcx antibody, which recognizes each of the four Lhcx isoforms. The upper part, with a Rubisco antibody, serves as loading control. **(B)** NPQ measurements. Wild type in black, x2KO_a in red, x2KO_b in pink, and the complemented strain x2KO_a + x2 in light blue. Low-light acclimated cells were concentrated to 10 mg Chl *a* L^–1^, exposed to 1,700-μmol photons m^–2^ s^–1^ for 10 min (a white bar), followed by 18 min of low light (a gray bar). Each experiment was performed in three or four biological replicates. SD is given. Data for preparing the graph for wild type were taken from Buck et al. (2019).

To investigate whether the CRISPR-Cas-based knockout approach was successful, we first cultivated the strains in an iron-depleted medium for 7 days before harvesting them for western blots, as iron depletion strongly affects Lhcx2 expression (Taddei et al., 2016). Lhcx2 was detectable in iron-starved wild type cells, but not expressed under nutrient-replete low-light conditions nor under any condition in the two x2KO strains (Figure 1A) created by either the TALEN or CRISPR-Cas approach. In the x2KO_a + x2 strain, a very modest expression of Lhcx2 was observed under nutrient-replete condition, while Lhcx2 was strongly upregulated in response to iron starvation, comparable to the wild type. This indicates that the introduced *Lhcx2* construct in x2KO_a + x2, regulated by the native *Lhcx2* promoter, responds similarly to iron-limiting conditions as the original gene in the wild type.

Under nutrient replete and low-light conditions, the overwhelming part of qE is provided by Lhcx1 (Buck et al., 2019). Consequently, we observed no relevant differences in NPQ capacity between wild type and the three mutated strains under these growth conditions (Figure 1B). If at all, there was a slightly lower NPQ capacity visible in both x2KO strains, probably resulting from some minor *Lhcx2* gene expression even under low-light conditions in wild type and x2KO_a + x2 (Figure 1A; Taddei et al., 2016).

Besides Lhcx proteins, the de-epoxidation state (DES) of the xanthophyll cycle and possibly, also the total xanthophyll cycle pool size, are important factors for qE (Olaizola et al., 1994; Lavaud et al., 2004; Goss et al., 2006; Schumann et al., 2007). To test the regulation of these pigments, we measured the xanthophyll cycle pool size and the DES in wild type and in x2KO_a in iron-replete conditions after a 2-h high light treatment as well as after following a 30-min recovery phase in low light (Supplementary Figure 2). In a second approach, we added cycloheximide (CHX) as an inhibitor of protein translation of nuclear encoded genes (such as Lhcx). Two hours of high light led to an upregulation of Lhcx2 in wild type, which is blocked by CHX (Lepetit et al., 2013; Buck et al., 2019). The total xanthophyll cycle pool size was increased in all experiments after high light treatment (e.g., ∼84/142-mmol/mol Chl *a* before/after high light treatment in wild type). An increased DES could be observed whenever CHX was added (e.g., ∼73%/55% DES with/without CHX in wild type). Due to the binding of Dd by Lhcfs and Lhcrs as well as, presumably, by Lhcx (Wang et al., 2019; Buck et al., 2021), the reduced Lhcx protein expression (due to CHX treatment) and the concomitant degradation of Lhcf and Lhcr under high light (Nymark et al., 2009) likely led to a larger pool of free Dd, which is not bound to any protein. Such lipid-dissolved Dd is de-epoxidized faster to Dt by the diadinoxanthin de-epoxidase (Schumann et al., 2007; Lepetit et al., 2010).

### An Expression Pattern of Iron-Induced Genes as Iron-Starvation Markers

**FIGURE 2:**
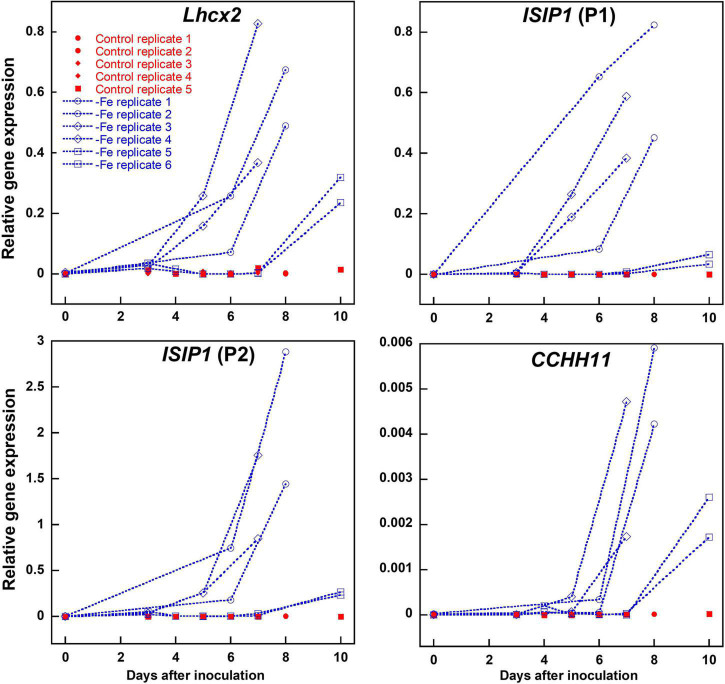
Comparison of *Lhcx2* expression with expression of *ISIP1* and *CCHH11* in an iron-depleted medium in *Phaeodactylum tricornutum* wild type cells. *ISIP1* was tested with two different primer pairs (P1 and P2) targeting the same transcripts. Measurements under iron limitation are shown in blue. Controls shown in red were treated the same way, but iron was added in a concentration like in a standard F/2 medium. Expression rates were normalized to expression of *18S*. Points with the same shape are technical replicates inoculated from the same culture.

To obtain a reliable proof for intracellular iron limitation, we investigated gene regulation of *iron-starvation*-*induced protein 1* (*ISIP1*) and *C2H2 zinc finger family protein CCHH11*, which in previous transcriptomic analyses were identified to be among the first genes being upregulated upon internal iron limitation (Allen et al., 2008; Smith et al., 2016). *ISIP1* was amplified by two different primer combinations independently. We also analyzed *Lhcx2* expression, previously shown to respond to iron limitation besides high light exposure (Taddei et al., 2016). All three genes were strongly upregulated almost simultaneously within 5–10 days after inoculation in an iron-depleted medium (Figure 2). In experiments where expression of *Lhcx2* started later or earlier than the average, expression of *CCHH1* and *ISIP1* was shifted similarly (e.g., Figure 2; replicates 5 and 6).

Marker gene expression correlated linearly with the expression of *Lhcx2*, with *R*^2^ values of 0.770 [*ISIP1* (P1)], 0.826 [*ISIP1* (P2)], and 0.861 (*CCHH11*) (Supplementary Figure 3). We performed a Spearman rank-order correlation test (Supplementary Table 1) in which *Lhcx2* expression correlates better with *ISIP1* [correlation coefficients (CC) of 0.895 and 0.913] than with *CCHH11* (CC = 0.753). All correlations are significantly different from zero with *p*-values < 0.001.

### Physiological Changes in Iron Limitation

#### Non-photochemical Fluorescence Quenching

**FIGURE 3:**
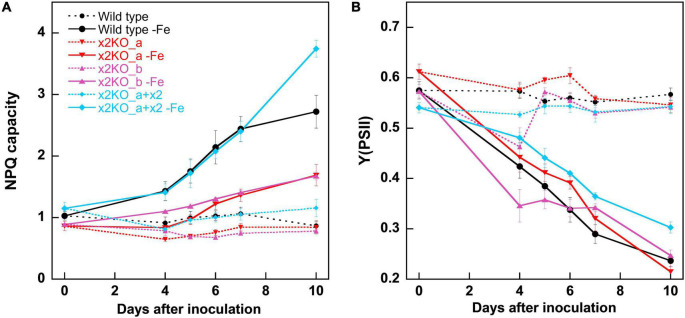
Non-photochemical fluorescence quenching (NPQ) capacities **(A)** and Y (PSII) **(B)** within 10 days after transfer of *Phaeodactylum tricornutum* wild type and mutant strains to an iron-depleted medium. For NPQ measurements, cells were exposed to ∼500-μmol photons m^–2^ s^–1^ of blue light in an Imaging-PAM. Mean values are derived from three to six independent replicates. SE is given.

Iron limitation is known to influence multiple cellular parameters. Some of the changes, especially the rise in the qE capacity, were assumed to result from upregulation of Lhcx2 (Taddei et al., 2016; Lepetit et al., 2017), which we tested using the Lhcx2-KO mutants. Within 10 days in the iron-depleted medium, the NPQ capacity of wild type was enhanced from ∼1.0 to ∼2.7, especially between the fifth and the seventh days after inoculation (Figure 3A). This happened concomitantly with the rise in *Lhcx2* transcripts (Figure 2). In both Lhcx2-KO lines, the rise in NPQ was much weaker, changing from 0.9 at Day 0 to ∼1.5 at Day 10. In x2KO_a + x2, the rise in NPQ was even more prominent than in the wild type (∼1.2 to ∼3.7) (Figure 3A). Thus, Lhcx2 is of major importance for the adjustment of qE under iron-limiting conditions.

#### Photosynthetic Yield

The photosynthetic yield of PSII [Y(PSII) or F_v_/F_m_] in the classical view describes the proportion of the absorbed energy, which can be maximally used by PSII for charge separation. Recently, this explanation has been challenged, as it was shown that the maximum fluorescence values can only be obtained by multiple turnover flashes with sufficient waiting times in between. This induces conformational changes within PSII, leading to a so-called light-adapted charge-separated state (Sipka et al., 2021). Therefore, this widely used parameter cannot be considered to assess the maximal photosynthetic yield anymore. For comparison of different physiological states, one can still rely on this parameter, and the strong decrease of Y(PSII) is very characteristic for iron starvation (Greene et al., 1991; Behrenfeld and Milligan, 2012). In our experiments, Y(PSII) gradually decreased during 10 days after inoculation in the iron-depleted medium in all strains (Figure 3B), reaching values of ∼0.25 in wild type and the two x2KO lines and slightly higher values in the complemented strain. The decrease of Y(PSII) slightly preceded the increase in iron-responsive gene expression of *Lhcx2*, *ISIP1*, and *CCHH11*, indicating that cells first experience slight changes in photophysiology due to minor decreases in internal iron content before they invest in expression of iron-responsive genes.

#### Pigment Composition

**FIGURE 4:**
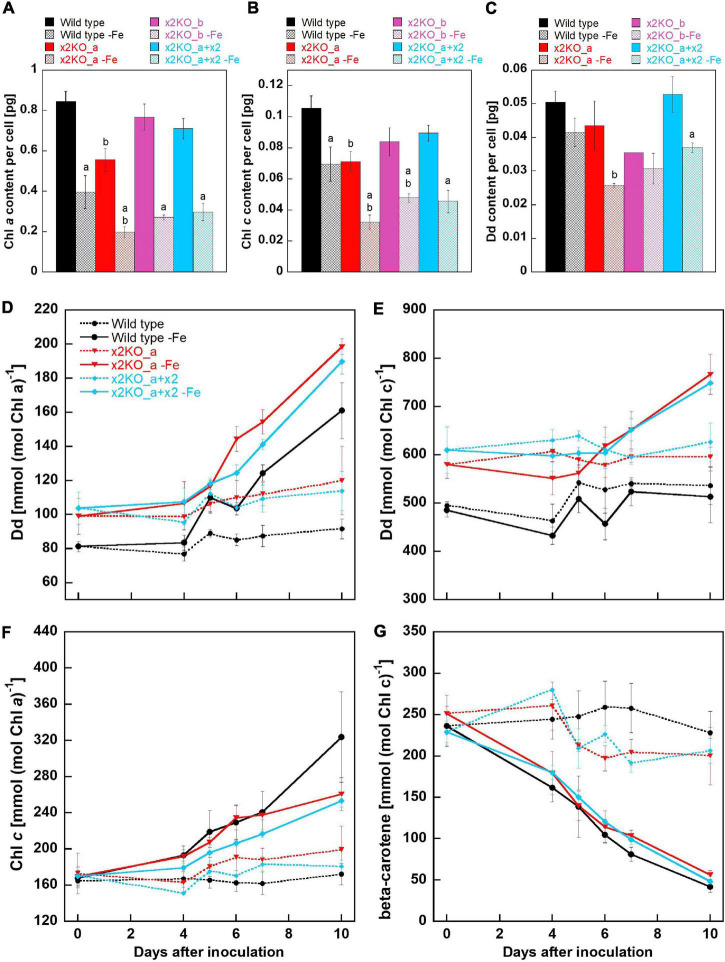
Pigment analysis of *Phaeodactylum tricornutum* wild type and mutant strains within 10 days after transfer to an iron-depleted medium. **(A)** Chl *a* amount per cell. **(B)** Chl *c* amount per cell. **(C)** Dd amount per cell. Mean values are derived from three to seven independent replicates except for Dd content/cell of the x2KO_b line (one replicate) and of the x2KO_b-Fe line (two replicates). SE is given. Statistical significance was tested by a two-tailed unpaired Student’s *T*-test (except for the x2KO_b and x2KO_b-Fe lines in C): (a) statistically significant in iron-replete vs. iron-depleted strain; (b) statistically significant in the respective mutant vs. the wild type exposed to the same iron treatment. **(D)** Dd content normalized to Chl *a*. **(E)** Dd content normalized to Chl *c*. **(F)** Chl *c* content normalized to Chl *a*. **(G)** β-carotene content normalized to Chl *c*. Mean values are derived from three to seven independent replicates. SE is given.

Previously, it has been demonstrated that iron depletion results in reduced amounts of pigments per cell, especially in Chl *a* (Allen et al., 2008; Lommer, 2012). Indeed, in all strains, the amount of Chl *a* per cell was significantly reduced (more than 50%) after 10 days in the iron-depleted medium, but there was no statistically significant difference in Chl *a*/cell between wild type, x2KO_b, and the complemented line under iron limitation (Figure 4A). However, the x2KO_a showed a lower Chl *a*/cell amount than the wild type both under iron-replete and iron-depleted conditions, which was not expected (especially under iron-replete condition, where Lhcx2 expression is negligible). We, therefore, assume that in this strain, there may be an additional mutation as a side effect of introducing the two TALEN plasmids by biolistic transformation for generating this mutant (cf. Buck et al., 2019). When calculating the amount of Chl *c* per cell, a significant reduction under iron limitation was also visible in all strains, albeit not as pronounced as the reduction in Chl *a* (Figure 4B). Regarding the xanthophyll cycle pigments, only Dd was detected, as low light conditions during growth did not lead to any de-epoxidation under iron limitation. Here, a tendency for a lower Dd content per cell was visible in all strains, which, however, could be statistically supported only in the complemented line (Figure 4C). In sum, Chl *a*, Chl *c*, and Dd decreased during iron limitation, albeit to different extents.

To obtain a refined picture of Dd dynamics, and to better resolve relative changes per available pigments in the plastid, we measured the pigment content over the whole period of 10-day iron starvation and normalized on Chl *a* and Chl *c*, respectively. Chl *a* is present within the photosystems and in the peripheral antenna, the Fucoxanthin Chlorophyll binding Proteins (FCPs). In contrast, Chl *c* is only bound in the FCPs and, therefore, allows normalization exclusively to the size of the peripheral antenna. During iron limitation, the amount of Dd per Chl *a* approximately doubled in all three strains within 10 days (although it decreased per cell, see above), while it remained at a similar level in iron-replete conditions (Figure 4D). However, by normalizing Dd to Chl *c*, no difference between the replete- and iron-limited wild type cells could be observed until Day 7, while slightly enhanced Dd levels were found in x2KO_a and x2KO_a + x2 after 10 days (Figure 4E). The discrepancy between Chl *a* vs. Chl *c* normalized data in all three strains results from a stronger reduction in photosystems compared to the reduction in FCPs. This is visualized by the stronger decrease in Chl *a*/cell than Chl *c*/cell (Figures 4A,B) and by the increase of Chl *c* normalized to Chl *a* after shifting cells in the iron-depleted medium (Figure 4F). The change in the ratio of peripheral antenna to photosystems is even more highlighted by measuring β-carotene. β-carotene is bound exclusively in the photosystems and not in the peripheral antenna. We observed a gradual reduction of β-carotene/Chl *c* up to ∼80–90% after 10 days (Figure 4G), indicating that major parts of the photosystems had been decomposed under iron limitation.

#### Functional Absorption Cross Section of Photosystem II

**FIGURE 5:**
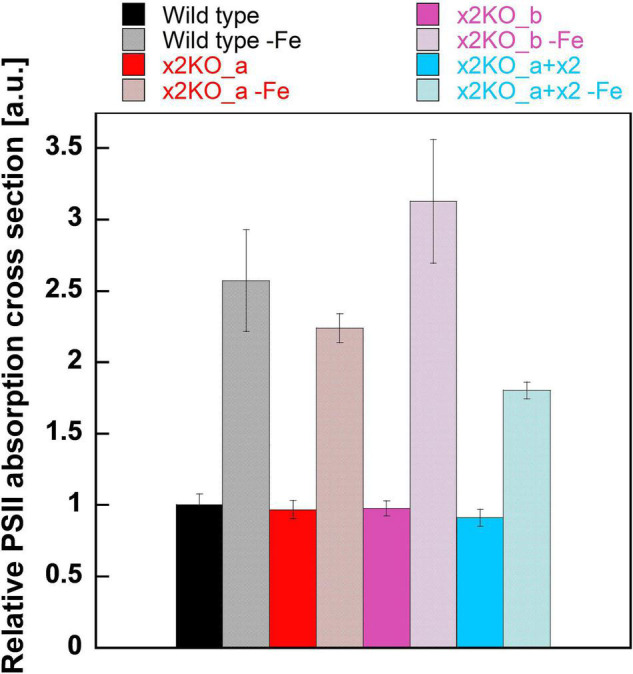
Functional absorption cross section of PSII (σPSII) in iron-replete and iron-limited *Phaeodactylum tricornutum* wild type and mutant strains. To measure σPSII, dark-acclimated cells were treated with DCMU and exposed to a red-light flash after 5 s (for details, refer to Section “Materials and Methods”). Values are normalized on the relative PSII functional absorption cross section of wild type cells grown in an iron-replete medium. Mean values are derived from three to six independent replicates. SE is given.

To obtain insights into possible changes in functional antenna size of PSII in iron-limited cells, we measured the relative functional absorption cross section of PSII (σPSII). σPSII of nutrient-replete strains was identical in all cases (Figure 5). After a 10-day cultivation in the iron-depleted medium, σPSII rose strongly, reaching values approximately two to three times of the initial values in replete cells. Therefore, the functional antenna size of each PSII complex was strongly increased, despite the reduced total Chl *a* content per cell. In line with the pigment data (cf. Figure 4), this indicates a massively reduced number of PSII per cell, with each one having a larger peripheral antenna.

#### 77 K Fluorescence Emission Spectra

**FIGURE 6:**
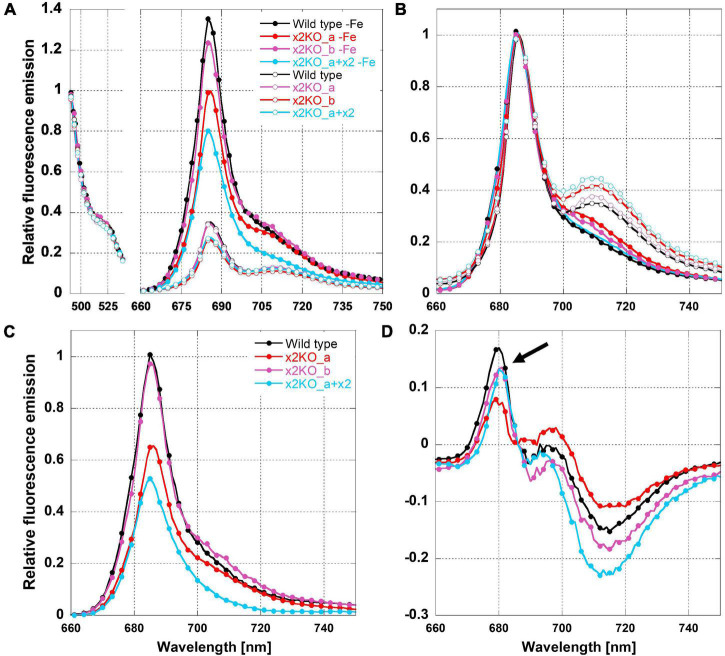
Relative 77K fluorescence emission spectra of iron-limited and replete *Phaeodactylum tricornutum* wild type and mutant strains. **(A)** Spectra normalized to the emission peak of green fluorescence protein (GFP), which was added as an internal standard. **(B)** Spectra normalized to the emission peak of Chl *a* at 686 nm. **(C)** Difference of 77 K fluorescence emission spectra of iron-deficient minus iron-replete cells normalized to the GFP emission. **(D)** Difference of 77 K fluorescence emission spectra of iron-deficient minus iron-replete cells normalized to the Chl *a* fluorescence emission peak at 686 nm. The arrow indicates fluorescence emission of FCPs not connected to the photosystems.

To determine possible influences of Lhcx2 on the presence of decoupled antenna complexes under iron limitation, we measured 77 K fluorescence emission spectra of iron-replete and iron-depleted cells (Figure 6). PSII-complexes of *P. tricornutum* emit fluorescence peaking at ∼686 nm, while the fluorescence emission of PSI-complexes peaks at ∼710–715 nm (Lepetit et al., 2007; Lepetit, 2010; Herbstová et al., 2015).

We adjusted strains to the same Chl *a* concentration and added a superfolding green fluorescence protein (GFP) as an internal standard. The fluorescence emission of Chl *a* was either normalized to the emission of GFP at 493 nm (Figure 6A) or to the peak fluorescence of Chl *a* at 686 nm (Figure 6B). Combined with the equal Chl *a* concentration, GFP normalization allows direct comparison of fluorescence intensities per Chl *a* between the different acclimation states, which, otherwise, is difficult in 77 K experiments due to non-homogeneous freezing processes. As expected, the 77 K fluorescence yield in iron-depleted cells was strongly increased at the PSII peak, likely due to the presence of uncoupled FCPs (Figure 6A; Greene et al., 1991; Behrenfeld and Milligan, 2012), as well as a much lower packing effect because of the reduced amount of Chl *a*/cell (Dubinsky and Schofield, 2010).

The presence of uncoupled antennae is better visualized in difference spectra calculated from 686-nm normalized fluorescence spectra of iron-deficient minus iron-replete cells (Figure 6D). The resulting maximum at ∼680 nm is derived from the emission of free FCPs that have a maximum shifted to shorter wavelengths compared to PSII supercomplexes (Lepetit, 2010; Büchel, 2015; Schaller-Laudel et al., 2015). We have to note that the fluorescence emission of these unconnected FCP complexes was relatively low (Figure 6D). This indicates that only a minor part of antenna complexes is detached during iron depletion in line with the massive increase in functional PSII absorption cross section (Figure 5).

In iron-replete cells, the PSI peak was clearly visible at ∼710 nm (Figure 6B). In contrast, in iron-depleted cells, the 710-nm fluorescence was barely detectable as a peak but rather as a shoulder. This indicates a reduction in PSI. For determining a PSII:PSI ratio, a quantitative comparison of the 686 and 710-nm peaks in iron-depleted cells is not possible because disconnected FCP complexes may at least partly add fluorescence to the 686-nm peak.

### Reactive Oxygen Formation in the Photosynthetic Apparatus

**FIGURE 7:**
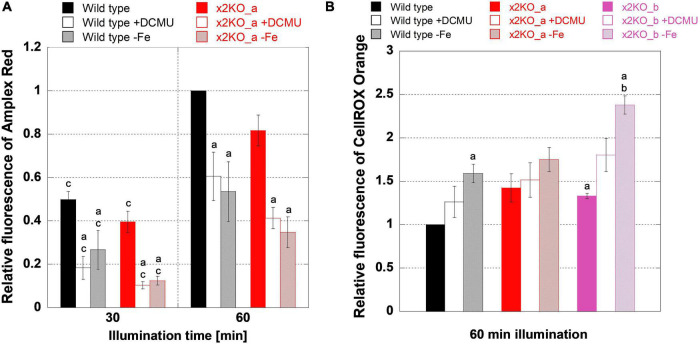
Reactive oxygen species measurements during high light treatment. Low-light-adapted cells were adjusted to 5-μg ml^–1^ Chl *a* and treated with 10-μM Amplex Red for H_2_O_2_ measurements or 25-μM CellROX Orange for general oxidative stress measurements prior to 1 h of high light exposure with 500-μmol photons m^–2^ s^–1^. **(A)** H_2_O_2_ measurements in iron-replete wild type and x2KO_a cells, without and with DCMU, and after 10 days of cultivation in the iron-depleted medium. **(B)** General oxidative stress measurement in iron-replete wild type, x2KO_a, and x2KO_b cells, without and with DCMU, and cells after 10 days of cultivation in the iron-depleted medium. Fluorescence intensities were normalized to wild type after 60 min for each experimental setup. Mean values are derived from 3 to 13 independent replicates. SE is given. Significance was tested by a two-tailed unpaired Student’s *T*-test: (a) statistically significant in the treated vs. the respective untreated strain; (b) statistically significant in the mutant(s) vs. the wild type exposed to the same treatment; (c) statistically significant in the same strain exposed to the same treatment but comparing two time points.

Reactive oxygen species might be formed more intensively in response to iron limitation but, here, might be alleviated by qE. To get first insights into ROS formation under iron-replete conditions, we treated cells with high light of 500 μmol photons m^–2^s^–1^ for 1 h, which is sufficient to saturate photosynthesis and activate qE (Figure 3A). First, by applying an Amplex Red assay, we tested how much H_2_O_2_ is formed. During a 1-h high light treatment, the amount of H_2_O_2_ significantly rose in wild type and x2KO_a, doubling from time point 30 min to time point 60 min (Figure 7A). No significant differences in H_2_O_2_ content between the two strains were observed. H_2_O_2_ may result from three major sources in diatom cells: the mitochondrial respiration, the photosynthetic Mehler reaction, and from a step of photorespiration in the peroxisomes. To block the photosynthetic sources (Mehler reaction and, indirectly, photorespiration), we treated cells with DCMU, which inhibits the linear photosynthetic electron transport by binding at the Q_B_-binding site of PSII. Cells, indeed, produced significantly lower amounts of H_2_O_2_ compared to untreated samples (Figure 7A), which indicates that, in high light, a significant proportion of H_2_O_2_ originates from electrons of the photosynthetic electron transport chain and/or photorespiration.

Besides H_2_O_2_ (and its precursor superoxide), which originates primarily at the acceptor side of PSI, ^1^O_2_ generated at PSII is the other major ROS in the chloroplast. It creates various oxidation products including lipid peroxides (Halliwell, 2006; Triantaphylidès and Havaux, 2009; Dietz et al., 2016). To measure more general oxidative stress levels, we used CellROX Orange, which is known to react with various ROS or their reactions products (Bone and Seredick, 2013; Celeghini et al., 2019; Escada-Rebelo et al., 2020). Many of these ROS or reaction products are formed because of the Mehler reaction and, therefore, should be affected by DCMU in a similar way like H_2_O_2_.

However, in contrast to the Amplex Red experiments, DCMU did not lower ROS formation detected by CellROX Orange in all strains (Figure 7B) despite the strongly reduced H_2_O_2_ (Figure 7A). There was also a significantly higher ROS production in the x2KO_b with and without DCMU treatment compared to wild type. The x2KO_a strain showed a tendency to produce more ROS compared to wild type, which failed statistical significance support marginally though (*p* = 0.082). Therefore, in contrast to H_2_O_2_ formation, the capacity of qE might have an effect on general ROS production. As Lhcx2 is upregulated immediately upon high light stress (Lepetit et al., 2013), there is a different amount of qE expected between wild type and the two x2KO lines during the period of high light incubation. Moreover, under our growth conditions, both x2KO lines already started with a slightly lower qE capacity (cf. Figure 1B).

We then analyzed H_2_O_2_ levels by Amplex Red in iron-depleted cells. H_2_O_2_ levels were similar between wild type and x2KO_a (Figure 7A). Also, H_2_O_2_ levels rose from 30- to 60-min high light in a similar way under both iron-replete and iron-depleted conditions, consequently approximately doubling from the 30-min values. This means that H_2_O_2_ formation was fully independent of a low or high qE capacity also under iron-depleted conditions. Interestingly, at both time points (30- and 60-min high light exposure), significantly reduced H_2_O_2_ levels were observed in iron-depleted cells compared to the replete controls. Worth noting is that the amount of H_2_O_2_ formed under iron depletion is highly comparable to that formed under iron-replete conditions when blocking photosynthesis by DCMU.

We also investigated total ROS production with CellROX Orange in iron-depleted cells. In contrast to the strongly reduced H_2_O_2_ levels, iron-depleted strains showed more ROS after high light exposure compared to the nutrient-replete strains, which was significant for wild type and the x2KO_b line (Figure 7B). The latter line had also a higher ROS level than the wild type under iron-deplete conditions. Both effects were also visible for the x2KO_a line, albeit not being statistically supported. In contrast to the Amplex Red experiments, iron limitation showed a tendency to increase general ROS production compared to DCMU treatments, which at least, in the x2KO_b line, was close to be statistically supported (*p* = 0.057). Here, however, experiments with many more replicates would be needed to allow a definite conclusion.

## Discussion

### *Lhcx2* Is Similarly Regulated as Classical Iron-Responsive Genes

Removing iron from the medium does not immediately lead to cellular iron shortage, but it is difficult to monitor intracellular iron stress. Classical physiological parameters like the F_v_/F_m_ decrease are one indicator of iron limitation and proved to be valuable also in our experiments. However, we sought for an additional, more specific indicator of internal iron limitation. Previously, Allen et al. (2008) and Smith et al. (2016) reported that *ISIP1* and *CCHH11* transcripts are highly upregulated in iron-limited *P. tricornutum*. ISIP1 is involved in a high affinity uptake system of iron-chelating siderophores (Kazamia et al., 2018). Thus, *ISIP1* is upregulated in the moment the cells suffer from internal iron limitation and, hence, serves as a suitable marker gene to monitor iron limitation. *ISIP1* is not present in all diatom species, but it is proposed to be of key importance, especially for oceanic species to cope with iron limitation (Kazamia et al., 2018). As far as we know, the function of CCHH11 has not been investigated yet, but it was annotated as a zinc-finger transcription factor probably involved in triggering gene expression in response to iron limitation.

We tracked the transcription of *Lhcx2*, *ISIP1*, and *CCHH11* in wild type growing for 10 days in an iron-depleted medium (Figure 2). All three genes were strongly upregulated, especially from the fifth to the seventh days, similarly to results obtained by Kazamia et al. (2018), and their expression correlated linearly and statistically significant. Thus, *Lhcx2* can serve as another marker of iron limitation, given the light intensities during growth remain the same. The similar expression pattern of *Lhcx2*, *ISIP1*, and *CCHH11* indicates that the same signaling pathway regulates their expression, at least under iron starvation and further indicates that the cells have generally shifted to an “iron limitation mode.” Indeed, the upregulation of these genes correlates well with the decrease of F_v_/F_m_ and an increase of NPQ capacity, both indicative of iron limitation (Behrenfeld and Milligan, 2012; Schuback et al., 2015; Taddei et al., 2016).

### Lhcx2 Is an Important Factor in Quenching Under Iron Limitation

An enhancing effect of Lhcx2 on qE capacity was previously shown by Buck et al. (2019), by overexpression studies of Taddei et al. (2016), and by correlating the rise in qE during prolonged high light treatment with protein expression of Lhcx2 (Lepetit et al., 2017). Under iron limitation, a larger qE capacity was suggested to be based on increased Lhcx2 expression (Taddei et al., 2016).

In our iron limitation experiments, the NPQ capacity rose strongly in wild type cells, but only to a small degree in the x2KO strains (Figure 3A). In accordance with Taddei et al. (2016), this increase occurs in parallel to the upregulation of *Lhcx2* (Figure 2). These results, combined with the overcompensated NPQ in x2KO_a + x2, proof that Lhcx2, indeed, is of key importance for the acclimation of qE capacity during iron limitation (Figure 3A). Enlargement of the xanthophyll cycle pool was not involved in this increase because we could not measure relevant differences in the pool size normalized to Chl *c* (Figure 4C) and thus no increase of xanthophyll cycle pigments in the peripheral antenna, where qE is located (Kuzminov and Gorbunov, 2016; Buck et al., 2019).

Even though *Lhcx2* is the only relevantly upregulated Lhcx isoform in *P. tricornutum* (Taddei et al., 2016), the iron-limited x2KO strains still exhibited some enhanced NPQ capacities compared to the nutrient-replete cells (Figure 3A). NPQ contains other components besides qE, in diatoms predominantly qI, which, generally, is regarded as a result of photoinhibition at PSII (Falkowski and Raven, 2013). Depending on the intensity of illumination and the qE capacity, qI can represent a relatively large proportion of total NPQ. This is visible in qE-deficient Lhcx1-knockout mutants, which almost exclusively possess qI (Buck et al., 2019). qI is probably increased in iron-limited cells due to a number of reasons: The PSII functional antenna size is relatively enlarged under iron limitation (Figure 5; Greene et al., 1991; Petrou et al., 2014; Trimborn et al., 2019), predominantly by reduced photosystem core components (Figure 4; Greene et al., 1991; Kosakowska et al., 2004; van Creveld et al., 2016). Also, the capacity of the photosynthetic electron transport chain is additionally limited by pronounced downregulation of cytochrome c_6_, the electron carrier between cytb_6_f and PSI (Roncel et al., 2016). Hence, a higher number of photons is harvested per functional reaction center, which results in a higher ETR relative to the number of PSII (Schuback et al., 2015), enhancing excitonic pressure and thus favoring qI. Besides, as reported elsewhere (Greene et al., 1991; Kosakowska et al., 2004; Allen et al., 2008; Lommer, 2012) and also observed in our experiments, the pigmentation per cell, and, therefore, the total antenna size per cell is reduced under iron limitation (Figure 4A) while expression of *Lhcx1* and *Lhcx3* is relatively unaffected (Taddei et al., 2016). Both, Lhcx1 and Lhcx3 also provide qE capacity (Buck et al., 2019). This suggests a higher Lhcx:FCP ratio in iron-limited cells, which might lead to some additional increase of qE capacity in all strains independent of Lhcx2.

### Lhcx2 Does Not Lead to a Detachment of Antenna Complexes During Low Light and Iron Limitation

Reduced Y (PSII) can be observed in iron-limited photosynthetic organisms like *P. tricornutum* (Behrenfeld et al., 2006; Allen et al., 2008; Behrenfeld et al., 2009), probably as a result of a pool of uncoupled antenna proteins (Greene et al., 1991; Behrenfeld and Milligan, 2012). Lhcx-mediated qE is proposed to involve physical detachment of antenna proteins from PSII cores, leading to a reduction in σPSII (Miloslavina et al., 2009; Chukhutsina et al., 2014; Derks et al., 2015; Goss and Lepetit, 2015; Giovagnetti and Ruban, 2017; Buck et al., 2019). This effect on σPSII has been demonstrated for all three Lhcx proteins involved in qE (Buck et al., 2019). We, therefore, tested whether Lhcx2 is possibly involved in a similar uncoupling mechanism during iron limitation even under low light, which hence, would be independent of the de-epoxidation of Dd to Dt.

In iron-limited cells, the Y(PSII) level (Figure 3B) gradually decreased in parallel to other parameters like the increase in qE capacity and the upregulation of the iron-induced genes including *Lhcx2* (Figures 2, 3A). Also, we observed the spectroscopic signature of unbound FCPs at 77 K. However, these effects were equally pronounced both in wild type and x2KO cells. Therefore, Lhcx2 has no structural effect on the general pigment protein complex structure in thylakoids under non-stressful light conditions during iron limitation.

The unbound FCPs might be disconnected from both PSI and PSII, because both photosystems reduce their abundance compared to the antenna fraction (Figures 4G, 5; Greene et al., 1991; Kosakowska et al., 2004; Lommer, 2012). As the fluorescence emission of both Lhcf (PSII antenna) and Lhcr (PSI antenna) proteins in diatoms is indistinguishable when not connected to the photosystems (Lepetit et al., 2007), the exact origin of the uncoupled FCPs could not be resolved.

### Impact of Iron Limitation on Xanthophyll Cycle Pigments

The de-epoxidation state of the xanthophyll cycle is essential for qE in diatoms (Lavaud et al., 2004; Goss et al., 2006; Buck et al., 2019; Lacour et al., 2019; Blommaert et al., 2021). The influence of the total pool size on qE capacity is, however, rather unclear (Lavaud et al., 2002b; Goss et al., 2006; Schumann et al., 2007; Lavaud and Lepetit, 2013). Importantly, the amount of de-epoxidizable Dd per number of available chlorophylls in the peripheral antenna is more decisive for qE capacity than the number of Dd per cell. In our iron limitation experiments, the xanthophyll cycle pool size—although decreasing per cell—approximately doubled when normalized to Chl *a* (Figure 4D), in line with Kosakowska et al. (2004), suggesting that an enlargement of the xanthophyll cycle pool contributes to increased qE capacity (Figure 3A). However, it has to be considered that iron limitation leads especially to a major reduction in Chl *a* and β-carotene containing photosystems, while the reduction in Chl *a* and Chl *c* containing FCPs is less pronounced (Greene et al., 1991). When normalized to Chl *c* and thus indirectly on the FCPs (Chl *c* is not found in the photosystem cores), we could not observe an enlarged xanthophyll cycle pool in wild type under iron limitation (Figure 4E). Therefore, a fixed ratio of xanthophyll cycle pigments per FCP is maintained.

Consequently, the rising NPQ capacities under iron limitation are primarily controlled by the available amount of Lhcx proteins rather than by changes in the xanthophyll cycle pool size. Probably, under most conditions with an increased xanthophyll cycle pool (e.g., Lavaud et al., 2002b; Schumann et al., 2007; Lepetit et al., 2017; Lacour et al., 2019), Lhcx content is also increased and the available amount of Dd is much higher than required to trigger qE by starting de-epoxidation. This is best exemplified by Lhcx1 overexpressing strains, showing a huge qE capacity (Lepetit et al., 2017), by various different natural *P. tricornutum* variants showing differences in Lhcx1 and NPQ (Bailleul et al., 2010), or by *P. tricornutum* cells grown in intermittent light, having an enormously high NPQ paralleled by massive increases in Lhcx (Giovagnetti et al., 2021). In all these strains and conditions, the total xanthophyll pool size did not change. The additional xanthophyll cycle pigments not involved in qE probably rather serve as antioxidants (Lepetit et al., 2010).

### Iron Limitation Affects Reactive Oxygen Species Formation

H_2_O_2_ formation was remarkably lower (∼50%) in iron-depleted conditions (Figure 7A), while the knockout of Lhcx2, despite the lower qE capacity of the x2KO mutants (Figure 3A), had no significant effect. This reduction in H_2_O_2_ occurred despite the previously demonstrated lower amount of major ROS-scavenging enzymes, including catalases and peroxidases (Allen et al., 2008; Smith et al., 2016; van Creveld et al., 2016). Reduced H_2_O_2_ formation is likely the result of the reduced PSI amount as well as decreased Cyt *c*_6_ levels under iron limitation (Allen et al., 2008; Behrenfeld and Milligan, 2012; Roncel et al., 2016; van Creveld et al., 2016), thus, less electrons are available to form H_2_O_2_ in the Mehler reaction. Moreover, iron limitation in diatoms leads only to a minor decrease in cellular protein amounts (Bozzato et al., 2021), suggesting that the Calvin cycle capacity may not decline to a similar extent as the photosynthetic light reaction capacity. Therefore, the rate-limiting step of photosynthesis may change from the Calvin cycle toward the photosynthetic light reaction (Behrenfeld and Milligan, 2012; Roncel et al., 2016). Similarly, the Mehler reaction is completely blocked by adding DCMU, which causes equally lower amounts of H_2_O_2_ formation as under iron depletion, with the residual H_2_O_2_ resulting almost exclusively from mitochondrial respiration. Based on these results, we consider the Mehler reaction, which even under replete conditions does not play a prominent role in diatoms (Bailleul et al., 2015), to be virtually absent in iron-limited cells. As iron-stressed cells massively reduced the amount of Chl *a*/cell (∼50%, Figure 4A) and all Amplex Red experiments had been performed on cells normalized to the same amount of Chl *a*, iron-starved *P. tricornutum* cells produce only ∼25% of H_2_O_2_ per cell compared to iron-replete cells.

Rather than influencing H_2_O_2_ levels, we expected qE to reduce ^1^O_2_ formation because qE dissipates energy within the peripheral antenna of PSII where ^1^O_2_ is formed in supersaturating light (Nishiyama et al., 2006; Triantaphylidès and Havaux, 2009; Buck et al., 2019). Detection of ^1^O_2_ in whole cells is not trivial. A previously described ^1^O_2_ measurement probe, Singlet Oxygen Sensor Green (SOSG, Flors et al., 2006), provided similar signals in an illuminated reaction buffer as in buffer-containing *P. tricornutum* cells in several test experiments we performed and, hence, was unsuitable to detect ^1^O_2_. We, thus, measured total ROS during high light illumination using the fluorescence reagent CellROX Orange. Interestingly, the total ROS level was independent from DCMU treatment. As DCMU prevents specifically all PSI-derived ROS (such as superoxide, hydrogen peroxide, or the hydroxyl radicals), CellROX Orange must detect additional ROS, which can only result from PSII processes independent of the electron transport chain (as this is null in DCMU conditions). These processes are mainly related to ^1^O_2_ formation and following oxidation processes, such as lipid peroxidation (Triantaphylidès and Havaux, 2009). Thus, total ROS production is similar in control (lower amount of ^1^O_2_ but more H_2_O_2_) and DCMU-treated samples (lower amount of H_2_O_2_ but more ^1^O_2_).

Under iron-depleted conditions, we observed increased total ROS production after light stress, in contrast to H_2_O_2_ production, when normalized to Chl *a*. Hence, there must be a strong increase of ^1^O_2_ production or derived products. In line with this, Roncel et al. (2016) previously observed higher lipid peroxidation rates under iron limitation, probably resulting from elevated ^1^O_2_ (Yalcinkaya et al., 2019). The remarkably larger σPSII (Figure 5) and the presence of uncoupled FCPs under iron limitation (Figure 6D) are factors supporting ^1^O_2_ formation in PSII supercomplexes and, possibly, in isolated FCPs. Given the decrease of PSII reaction centers, indicated by the increased σPSII, the strong decrease of β-carotene per Chl *c* and the reduced amount of reaction center protein D1 (Greene et al., 1991), under iron depletion, the overwhelming part of ^1^O_2_ is produced in the peripheral antenna and not in the PSII cores.

The increase in total ROS under iron depletion, primarily based on ^1^O_2_ and derivative products, is less than twofold compared to replete conditions, normalized on the same Chl *a* concentration. Of note, we can take this only as a rough proxy, as our total ROS probe, CellROX Orange, in contrast to Amplex Red, gives a prominent background fluorescence signal. As in iron-depleted conditions the Chl *a*/cell content dropped by ∼50%, these data indicate no major increase in total ROS production per cell. Thus, the massive readjustment of the photosynthetic machinery enforced by the lack of iron leads to a similar ROS burden for both iron-replete and iron-depleted cells.

When it comes to comparing total ROS formation depending on the extent of qE, there is statistical support for a higher ROS production in the x2KO_b line compared to the wild type under iron limitation. The same effect, albeit much weaker, is visible in replete conditions after 1 h of high light stress, where Lhcx2 is also upregulated and provides a higher qE capacity in wild type (Lepetit et al., 2013). This indicates an effect of qE on reducing ROS production (and here, mainly on ^1^O_2_) and would explain why *P. tricornutum* increases its qE capacity in response to iron limitation by expressing Lhcx2. In line with this, elevated ^1^O_2_ concentrations or products directly derived from increased ^1^O_2_ have been detected in qE-deficient lines of *Arabidopsis thaliana* (Roach and Krieger-Liszkay, 2012) and *Chlamydomonas reinhardtii* (Roach et al., 2020). Unfortunately, the higher ROS production under iron limitation, although visible in a dampened manner, is not statistically supported in the *P. tricornutum* x2KO_a mutant. We assume that an off-side effect created by the biolistic transformation with two vectors in x2KO_a, indicated by changed Chl *a* and *c*/cell ratios (Figures 4A,B and Section “Pigment Composition”), might dampen the signal. At this stage, our data indicate a putative influence of qE in lowering total ROS in high-light conditions, but this needs to be verified in future in-depth analyses, especially by creating and measuring double Lhcx1/Lhcx2-KO mutants, where the difference in qE between wild type and mutant would be even larger.

## Conclusion

Iron limitation leads to a massive adjustment of the whole photophysiology in *P. tricornutum*, resulting in an iron limitation mode phenotype. This includes a general decrease of pigments per cell, an increased functional photosystem II antenna size due to reduction of both photosystems, a reduction of F_v_/F_m_ due to the presence of free chlorophyll-binding proteins, decreased H_2_O_2_ production due to a reduced photosynthetic light reaction, and a higher amount of total ROS per Chl *a* but not per cell. An additional iron-starved phenotype is the upregulation of qE capacity by increased expression of Lhcx2. This increased qE capacity alone does not impact significantly most of the other observed phenomena under iron starvation but may alleviate the total ROS production under additional high-light stress. Obviously, NPQ regulation is only one of the various components of a sophisticated iron acclimation strategy. These components allow diatoms to survive such severe stress conditions in relatively good shape, waiting for the next iron input to start blooming again, thereby outcompeting all other phytoplanktons.

## Data Availability Statement

The original contributions presented in the study are included in the article/Supplementary Material, further inquiries can be directed to the corresponding authors.

## Author Contributions

JB, BL, and PK planned the experiments, evaluated the results, and wrote the manuscript. JB and MW performed ROS measurements. JB, AS, and BL produced the CRISPR-Cas Lhcx2 knockout strain. AS produced recombinant GFP. BL set up the 77K GFP measurement setup. JB performed the residual experiments. All authors contributed to the article and approved the submitted version.

## Conflict of Interest

The authors declare that the research was conducted in the absence of any commercial or financial relationships that could be construed as a potential conflict of interest.

## Publisher’s Note

All claims expressed in this article are solely those of the authors and do not necessarily represent those of their affiliated organizations, or those of the publisher, the editors and the reviewers. Any product that may be evaluated in this article, or claim that may be made by its manufacturer, is not guaranteed or endorsed by the publisher.

## Footnotes

1
http://crispor.tefor.net/
