# Supplementary material for: Impact of Lhcx2 on Acclimation to Low Iron Conditions in the Diatom Phaeodactylum tricornutum
Source: Front Plant Sci. 2022 Mar 16;13:841058. doi: 10.3389/fpls.2022.841058 (PMC8967352; doi:10.3389/fpls.2022.841058)
Supplement: Supplementary file 1 [file Data_Sheet_1.PDF]

Supplemental information

Allele 1

```
Wild type allele 1      1 ATGAAATTATCCTTGGCTATCCTTGCGCTTTGCGCCAGCACTAGTGCCGCTTTCGCTCCT  60
x2KO_b allele 1         1 ATGAAATTATCCTTGGCTATCCTTGCGCTTTGCGCCAGCACTAGTGCCGCTTTCGCTCCT  60

Wild type allele 1     61 TCTGTTTCCCAGAGGACGTCTGTCTCTCTCCGAGAATCATTGGACCCCACGGACTCCATG 120
x2KO_b allele 1        61 TCTGTTTCCCAGAGGACGTCTGTCTCTCTCCGAGAATCATTGGACCCCACGGACTCCATG 120

Wild type allele 1    121 TCGGAAGTGGAAGGCGCCGTGAAAGACGCGGCTCCCAAAGTCTCCGACCCTTTCGACAGC 180
x2KO_b allele 1       121 TCGGAAGTGGAAGGCGCCGTGAAAGACGCGGCTCCCAAAGTCTCCGACCCTTTCGACAGC 180

Wild type allele 1    181 CCTCGTGATCTTGCCGGAGTCGTCGCTCCTACCGGCTTTTTCGATCCGGCAGGCTTCGCT 240
x2KO_b allele 1       181 CCTCGTGATCTTGCCGGAGTCGTCGCTCCTACCGGCTTTTTCGATCCGGCAGGCTTCGCT 240

Wild type allele 1    241 GCCCGAGCCGATGCCGGAACCATGAAGCGTTACCGGGAAGCGGAAGTTACTCACGGACGT 300
x2KO_b allele 1       241 GCCCGAGCCGATGCCGGAACCATGAAGCGTTACCGGGAAGCGGAAGTTACTCACGGACGT 300

Wild type allele 1    301 GTGGGCATGATGGCCGTTGTCGGCTTTCTTGCGGGCGAAGCCGTCGAGGGATCGTCCTTT 360
x2KO_b allele 1       301 GTGGGCATGATGGCCGTTGTCGGCTTTCTTGCGGGCGAAGCCGTCGAGGGATCGTCCTTT 360

Wild type allele 1    361 CTCTTTGACTCGCAAGTCAGCGGACCCGCCATTACTCACCTCAACCAGATTCCTTCCATC 420
x2KO_b allele 1       361 CTCTTTGACTCGCAAGTCAG----------------------AACCAGATTCCTTCCATC 398

Wild type allele 1    421 TTTTGGATTCTCCTCACGGTGGGCATTGGTGCTTCCGAAGTCACGCGCGCTCAAATTGGT 480
x2KO_b allele 1       399 TTTTGGATTCTCCTCACGGTGGGCATTGGTGCTTCCGAAGTCACGCGCGCTCAAATTGGT 458

Wild type allele 1    481 TGGGTACGTATCGGGAGTTGTTGGTGCCAACAGTTTCGCTTTGTCCGTCTCTCACGCGTT 540
x2KO_b allele 1       459 TGGGTACGTATCGGGAGTTGTTGGTGCCAACAGTTTCGCTTTGTCCGTCTCTCACGCGTT 518

Wild type allele 1    541 CTTTTCCTTTCCATTCGTTTTCCAGGTTGAACCCGAGAACGTCCCACCGGGCAAGCCGGG 600
x2KO_b allele 1       519 CTTTTCCTTTCCATTCGTTTTCCAGGTTGAACCCGAGAACGTCCCACCGGGCAAGCCGGG 578

Wild type allele 1    601 TCTCCTCCGCGACGATTACGTCCCGGGTGACATTGGCTTTGATCCTCTCGGCTTGAAGCC 660
x2KO_b allele 1       579 TCTCCTCCGCGACGATTACGTCCCGGGTGACATTGGCTTTGATCCTCTCGGCTTGAAGCC 638

Wild type allele 1    661 TTCCGACGCGCAGGCTCTCAAATCGATCCAGACCAAAGAACTGCAGAGTAAGTCATTTGC 720
x2KO_b allele 1       639 TTCCGACGCGCAGGCTCTCAAATCGATCCAGACCAAAGAACTGCAGAGTAAGTCATTTGC 698

Wild type allele 1    721 TGTTGTTGCTGTTACTGTCGTCCTTGGTACTATCGTGTTTACAGTTAGTTCACTGCGTCA 780
x2KO_b allele 1       699 TGTTGTTGCTGTTACTGTCGTCCTTGGTACTATCGTGTTTACAGTTAGTTCACTGCGTCA 758

Wild type allele 1    781 GAGTGTACCGTGTCATACTGTTAATCCCACCAAACTAACCGATTGAATATAACTCGTTTT 840
x2KO_b allele 1       759 GAGTGTACCGTGTCATACTGTTAATCCCACCAAACTAACCGATTGAATATAACTCGTTTT 818

Wild type allele 1    841 CTTTGGCTAGACGGACGTCTCGCCATGTTGGCGGCGGCTGGGTGCATGGCTCAGGAATTG 900
x2KO_b allele 1       819 CTTTGGCTAGACGGACGTCTCGCCATGTTGGCGGCGGCTGGGTGCATGGCTCAGGAATTG 878

Wild type allele 1    901 GCCAACGGAAAGGGCATTCTCGAAAACCTTGGTCTCTAA 939
x2KO_b allele 1       879 GCCAACGGAAAGGGCATTCTCGAAAACCTTGGTCTCTAA 917
```

Allele 2

```
Wild type allele 2      1 ATGAAATTATCCTTGGCTATCCTTGCGCTTTGCGCCAGCACTAGTGCCGCTTTCGCTCCT  60
x2KO_b allele 2         1 ATGAAATTATCCTTGGCTATCCTTGCGCTTTGCGCCAGCACTAGTGCCGCTTTCGCTCCT  60

Wild type allele 2     61 TCTGTTTCCCAGAGGACGTCTGTCTCTCTCCGAGAATCATTGGACCCCACGGAATCCATG 120
x2KO_b allele 2        61 TCTGTTTCCCAGAGGACGTCTGTCTCTCTCCGAGAATCATTGGACCCCACGGAATCCATG 120

Wild type allele 2    121 TCGGAAGTGGAAGGCGCCGTGAAAGACGCGGCTCCCAAAGTCTCCGACCCTTTCGACAGC 180
x2KO_b allele 2       121 TCGGAAGTGGAAGGCGCCGTGAAAGACGCGGCTCCCAAAGTCTCCGACCCTTTCGACAGC 180

Wild type allele 2    181 CCTCGTGATCTTGCCGGAGTCGTCGCTCCTACCGGCTTTTTCGATCCGGCAGGCTTCGCT 240
x2KO_b allele 2       181 CCTCGTGATCTTGCCGGAGTCGTCGCTCCTACCGGCTTTTTCGATCCGGCAGGCTTCGCT 240

Wild type allele 2    241 GCCCGAGCCGATGCCGGTACCATGAAGCGTTACCGGGAAGCGGAAGTTACTCACGGACGT 300
x2KO_b allele 2       241 GCCCGAGCCGATGCCGGTACCATGAAGCGTTACCGGGAAGCGGAAGTTACTCACGGACGT 300

Wild type allele 2    301 GTGGGCATGATGGCCGTTGTCGGCTTTCTTGCGGGCGAAGCCGTCGAGGGATCGTCGTTT 360
x2KO_b allele 2       301 GTGGGCATGATGGCCGTTGTCGGCTTTCTTGCGGGCGAAGCCGTCGAGGGATCGTCGTTT 360

Wild type allele 2    361 CTCTTTGACTCGCAAGTCAGCGGACCCGCCATTACTCACCTCAACCAGATTCCTTCCATC 420
x2KO_b allele 2       361 CTCTTTGACTCGCAAGTCAGCGGACCCGCCAT-ACTCACCTCAACCAGATTCCTTCCATC 419

Wild type allele 2    421 TTTTGGATTCTCCTCACGGTGGGCATTGGTGCTTCCGAAGTCACGCGGGCTCAAATTGGT 480
x2KO_b allele 2       420 TTTTGGATTCTCCTCACGGTGGGCATTGGTGCTTCCGAAGTCACGCGGGCTCAAATTGGT 479

Wild type allele 2    481 TGGGTACGTATCGGGAGTTGTTGGTTCCAACAGTTTCGCTTTGTCCGTCTCTCACGCGTT 540
x2KO_b allele 2       480 TGGGTACGTATCGGGAGTTGTTGGTTCCAACAGTTTCGCTTTGTCCGTCTCTCACGCGTT 539

Wild type allele 2    541 CTTTTCCTTTCCATTCGTTTTCCAGGTTGAACCCGAGAACGTCCCACCGGGCAAGCCGGG 600
x2KO_b allele 2       540 CTTTTCCTTTCCATTCGTTTTCCAGGTTGAACCCGAGAACGTCCCACCGGGCAAGCCGGG 599

Wild type allele 2    601 TCTCCTCCGCGACGATTACGTCCCGGGTGACATTGGCTTTGATCCTCTCGGCTTGAAGCC 660
x2KO_b allele 2       600 TCTCCTCCGCGACGATTACGTCCCGGGTGACATTGGCTTTGATCCTCTCGGCTTGAAGCC 659

Wild type allele 2    661 TTCCGACGCGCAGGCTCTCAAATCGATCCAGACCAAGGAACTGCAGAGTAAGTCATTTGC 720
x2KO_b allele 2       660 TTCCGACGCGCAGGCTCTCAAATCGATCCAGACCAAGGAACTGCAGAGTAAGTCATTTGC 719

Wild type allele 2    721 TGTTGCTGCTGTTACTGTCGTCCATGGTACTATCGTGTTTACAGTTAGTTCACTGCGTCA 780
x2KO_b allele 2       720 TGTTGCTGCTGTTACTGTCGTCCATGGTACTATCGTGTTTACAGTTAGTTCACTGCGTCA 779

Wild type allele 2    781 GAGTGTACCGTGTCATACTGTTAATCCCACCAAACTAACCGATTGAATATAACTCGTTTT 840
x2KO_b allele 2       780 GAGTGTACCGTGTCATACTGTTAATCCCACCAAACTAACCGATTGAATATAACTCGTTTT 839

Wild type allele 2    841 CTTTGGCTAGACGGACGTCTCGCCATGTTGGCGGCGGCTGGGTGCATGGCTCAGGAATTG 900
x2KO_b allele 2       840 CTTTGGCTAGACGGACGTCTCGCCATGTTGGCGGCGGCTGGGTGCATGGCTCAGGAATTG 899

Wild type allele 2    901 GCCAACGGAAAGGGCATTCTCGAAAACCTTGGGCTCTAA 939
x2KO_b allele 2       900 GCCAACGGAAAGGGCATTCTCGAAAACCTTGGGCTCTAA 938
```

Fig S1: Sequences of the two *Lhcx2* alleles in x2KO_b.
Highlighted in blue is the binding site for the CRISPR-Cas construct. In red the deletions are shown. In allele 1 of *Lhcx2*, x2KO_b has a deletion of 22 bp and in allele 2 a deletion of 1 bp. Both deletions lead to a frameshift and knockout of the gene.

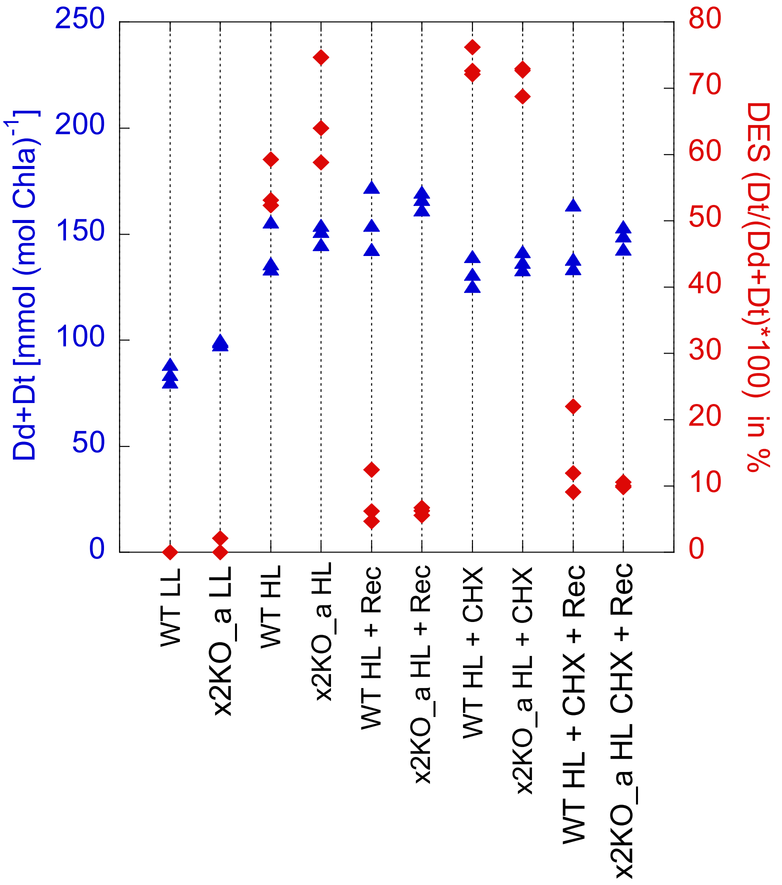

Fig. S2: Xanthophyll-cycle pigment pool size (Dd+Dt) and de-epoxidation state (DES) of wild type and x2KO_a.

Cells were low light acclimated, treated with 2 h of high light (HL; 1700 µmol photons m^{-2} s^{-1}) or 2 h HL treated followed by a 30 min recovery phase with 40 µmol photons m^{-2} s^{-1} (HL + Rec). Additionally, the same experiments were performed with 2 µg mL^{-1} cycloheximide (CHX) which was added before the illumination with the aim to block translation of nuclear encoded genes. Each experiment was performed in three biological replicates.

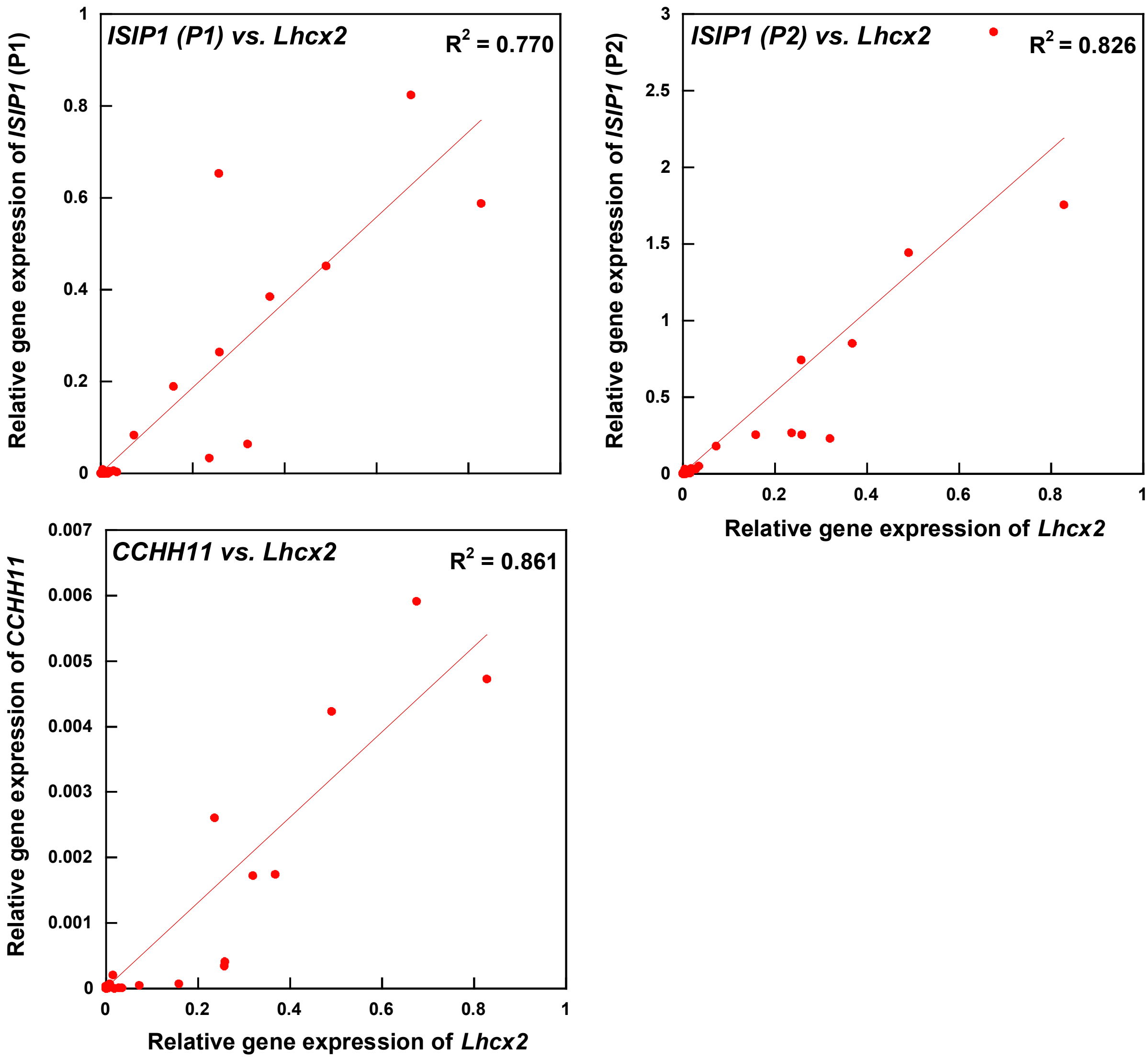

Fig. S3: Linear regression of the expression levels of *Lhcx2* and the two marker genes *ISIP1* and *CCHH11* under iron limitation.
The distributions of the expression levels suggest linear correlations of both marker genes with the expression of *Lhcx2*. n = 28.

Table S1: Correlation coefficients of the Spearman correlation tests between *Lhcx2*, *ISIP1* and *CCHH11* expression levels under iron limitation.
All of the three tested genes correlated positively with each other. *P* values are shown in brackets, indicating the probability of correlation. n = 28.

Gene	*ISIP1* (P1)	*ISIP1* (P2)	*CCHH11*
Lhcx2	**0.895** (p = 2.00 x 10^{-7})	**0.913** (p = 2.00 x 10^{-7})	**0.753** (p = 2.00 x 10^{-7})
***ISIP1* (P1)**		**0.983** (p = 2.00 x 10^{-7})	**0.689** (p = 2.95 x 10^{-6})
***ISIP1* (P2)**			**0.711** (p =2.96 x 10^{-6})
